# Syntactic prediction during self‐paced reading is age invariant

**DOI:** 10.1111/bjop.12594

**Published:** 2022-09-14

**Authors:** Michael G. Cutter, Kevin B. Paterson, Ruth Filik

**Affiliations:** ^1^ University of Nottingham Nottingham UK; ^2^ University of Leicester Leicester UK

**Keywords:** cognitive ageing, language comprehension, reading, self‐paced reading, syntactic prediction

## Abstract

Controversy exists as to whether, compared to young adults, older adults are more, equally or less likely to make linguistic predictions while reading. While previous studies have examined age effects on the prediction of upcoming words, the prediction of upcoming syntactic structures has been largely unexplored. We compared the benefit that young and older readers gain when the syntactic structure is made predictable, as well as potential age differences in the costs involved in making predictions. In a self‐paced reading study, 60 young and 60 older adults read sentences in which noun‐phrase coordination (e.g. *large pizza or tasty calzone*) is made predictable through the inclusion of the word *either* earlier in the sentence. Results showed a benefit of the presence of *either* in the second half of the coordination phrase, and a cost of the presence of *either* in the first half. We observed no age differences in the benefit or costs of making these predictions; Bayes factor analyses offered strong evidence that these effects are age invariant. Together, these findings suggest that both older and younger adults make similar strength syntactic predictions with a similar level of difficulty. We relate this age invariance in syntactic prediction to specific aspects of the ageing process.

## BACKGROUND

Many psychological processes have primarily been experimentally examined in university students under the age of 25, with less focus being given to other age groups, such as older adults above the age of 65. This includes research on the cognitive operations underlying the processing of written language, although an increased focus on the effects of ageing on these operations has emerged over the last 15 years (see Gordon et al., 2016; Paterson et al., 2020). This research contributes to the theoretical understanding of how cognitive processes in reading might change across the lifespan. However, it also has practical relevance, as being able to read well is important for older people to continue to function effectively in most modern societies, and to accomplish everyday tasks that enable them to live independently. With the current research, we examine a specific aspect of how changes in cognitive ability with increasing age may affect the reading process, by testing whether a reader's ability to make predictions about sentence structure and to adapt to novel linguistic environments alters across the adult lifespan. Recent theoretical approaches to language (e.g. Dell & Chang, 2014; Pickering & Gambi, 2018) have cast prediction as a central and fundamental part of the language comprehension process. Accordingly, gaining a clear understanding of how linguistic prediction may change with older age is vital to the more general issue of how language comprehension is affected by age.

Substantial research suggests that readers actively use contextual information, world knowledge, linguistic knowledge and other cues to make predictions about what might come next in a linguistic utterance, with these predictions aiding later processing (see Kuperberg & Jaeger, 2016; Pickering & Gambi, 2018, for recent reviews and theoretical frameworks). For example, a highly predictable word within a sentence (e.g. *bone* in *The dog buried his bone in the garden*) is identified more easily than an unpredictable word within that sentence (e.g. *food* instead of *bone*). This is demonstrated by the fact that readers respond more quickly to predictable words in a lexical decision task (e.g. Schwanenflugel & Shoben, 1985), spend less time looking at predictable words in eye‐movement studies and are more likely to skip these words (e.g. Cutter et al., 2020; Ehrlich & Rayner, 1981), and exhibit smaller electrophysiological responses to predictable words relative to unpredictable words (Kutas & Hillyard, 1984). However, it is unclear whether people's use of context to predict linguistic information changes during healthy adult ageing, with some investigations suggesting little change (e.g. Stine‐Morrow et al., 1999) and some suggesting either increased (e.g. Zhao et al., 2019, 2021) or decreased (e.g. Federmeier et al., 2010) context use with age (see Payne & Silcox, 2019, for a review). There also is disagreement concerning whether findings showing increased use of context by older adults demonstrate that this is being used predictively, to guide the processing of linguistic information; or, alternatively, to facilitate the integration of this information with the reader's unfolding interpretation of the text. Furthermore, different groups of researchers have put forward theoretical frameworks that assume either greater predictive use of context (e.g. Rayner et al., 2006) or reduced prediction in older adults (e.g. Federmeier et al., 2010) based on different aspects of the ageing process (e.g. reduced perceptual processing vs. reduced neuronal connectivity and executive functions).

The majority of work to date examining linguistic prediction and context use in older adults' reading has focused on the identification of individual words (e.g. Choi et al., 2017; Dave et al., 2018; Federmeier et al., 2010; Kliegl et al., 2004; Rayner et al., 2006; Steen‐Baker et al., 2017; Zhao et al., 2019, 2021), or the way in which a build‐up of discourse information aids reading (e.g. McKoon & Ratcliff, 2018; Miller et al., 2006; Stine‐Morrow et al., 1996; Valencia‐Laver & Light, 2000). However, recent studies of spoken language processing have used the visual world paradigm (Allopenna et al., 1998; Cooper, 1974) to investigate age differences in the predictive use of syntax to direct listeners' attention to the information in the visual environment, showing either no age difference in the use of this information (Baltaretu & Chambers, 2018) or possibly a small benefit for older adults (Huettig & Janse, 2016). Syntactic prediction arguably occurs at a level between word identification and discourse processing, and it is notable that no prior work has examined age differences in the predictive use of syntactic knowledge in reading. Accordingly, our aim with the present study was to take a first step in determining whether young and older adults differ in their use of reliable cues to predict upcoming syntactic structure while reading.

Specifically, we presented young and older adults with sentences including coordinated noun phrases (e.g. *Josh will order [either] a large pizza or a tasty calzone at the lunch*) which were rendered predictable by the presence of the word *either*, or left unpredictable due to its absence. When a verb is immediately followed by *either* and a noun phrase (e.g. *order either a large pizza…*), the only grammatical sentence continuation is for this first noun phrase to join with a subsequent noun phrase in a coordination structure, with the word *or* joining the two noun phrases (e.g. *…or a tasty calzone*). As such, *either* in this position allows readers to make strong predictions about upcoming syntactic constituents once they have also encountered the first noun phrase. It should be noted that noun‐phrase coordination is licit in the absence of *either*.

Staub and Clifton Jr. (2006) argued that the presence of *either* allows readers to build a coordination structure predictively, thus reducing processing demands on the reader upon reaching *or* and the second noun phrase. Specifically, when readers process coordination in the absence of *either* they should mistakenly treat the first noun phrase (e.g. *a large pizza*) as the only object of the verb (e.g. *order*), and must revise this analysis upon encountering *or a tasty calzone* (see Sturt & Lombardo, 2005). The argument put forward by Staub and Clifton Jr. (2006) claims that this difficulty occurs specifically due to readers (1) integrating *a tasty pizza* as an immediate constituent of *order* but then (2) having to remove this structure and instead insert a coordination structure upon encountering *or*. Predictively building a coordination structure prevents readers from making the initial mistake, thus removing this later processing effort. To be clear, this effect has specifically been attributed to the projection of upcoming syntactic structure upon encountering the first noun phrase after a verb followed by the word *either*, as opposed to it simply being the case that readers find it easier to integrate the second noun phrase into the sentence when it is preceded by *either*. We refer readers to the discussion of Staub and Clifton for a more in‐depth argument. However, it should be clear that, if this argument is correct, this scenario is ideally suited to testing age differences in the effects of syntactic prediction, as opposed to sentential integration, during reading.

Prior research has established that the presence of *either* can facilitate the processing of coordination structures by both young adults (Staub & Clifton Jr., 2006) and older adults (Warren et al., 2016). However, these two experiments were conducted independently, using two different methodologies (i.e. eye‐tracking vs. self‐paced reading) and a different set of stimulus items. Consequently, it is not possible to make a direct comparison between younger and older adults using the effects observed in these two experiments, and therefore to infer whether one age group made greater or lesser use of *either* in forming syntactic predictions. Accordingly, a principle goal of the present study was to establish whether young and older adults differ in their use of *either* to make syntactic predictions, using self‐paced reading to determine the size of the effect in both groups. This study, therefore, represents the first direct test of how ageing affects syntactic prediction during reading.

An investigation of age‐related differences in syntactic prediction is interesting for several reasons. While it is not entirely clear whether young and older adults differ in making linguistic predictions, several proposals exist as to why differences may occur. For example, Ryskin et al. (2020) recently outlined several mechanisms that may explain differences in linguistic prediction between different populations. These explanations fit broadly into two categories. The first relates to changes in executive function with age, encompassing a wide range of cognitive processes including working memory maintenance and updating, inhibitory control and set shifting (see, e.g. Friedman & Miyake, 2017; although it is important to note that other influential theories of cognitive ageing identify slowing in the speed with which cognitive operations can be executed as a further domain‐general variable that might affect language processing; Salthouse, 1996; see also Huettig & Janse, 2016). By comparison, the other explanation focuses on differences in language experience throughout the lifespan. Focussing on the latter explanation, Ryskin et al. argued that apparent adult age differences in predictive processing in many experiments might be due to the older adult participants making different predictions (but of the same amount/strength) compared to young adult participants. Such differences might reflect the different linguistic and world experiences of the two age groups. For example, whereas older adults might be more likely to predict the word *video* in the context ‘They were ready for the film so he played the…’, young adults might be more likely to predict *Blu‐ray* or *DVD*, with these responses reflecting differences in the specific world knowledge and experience of the two groups.

The use of *either* to predict upcoming structure provides an interesting test against whether age‐related changes in predictive processing can be wholly attributed to variations in language experience throughout the lifespan, as the above account might imply. As described earlier, when *either* appears directly before a noun phrase, the only legal sentence continuation upon encountering the first noun phrase is a noun‐phrase coordination structure. Presumably, once this rule is acquired by a developing reader, there is no further experience that should result in readers making weaker predictions upon encountering *either*. As such, a weaker effect for older adults in the present study would be problematic for accounts that attempt to explain age differences in prediction by appealing to differing levels of language experience across age groups, while equivalent or larger effects would be unproblematic for this account. It could be argued that accounts attributing reduced lexical prediction in older adults to declines in executive resources should expect similar declines in syntactic prediction. However, we will argue in the Discussion that this is not necessarily the case because of qualitative differences in the processes underlying lexical and syntactic prediction.

As well as examining whether young and older adults gain a similar level of benefit from cues to upcoming syntactic structure, we were interested in whether making these predictions is more difficult for one age group than the other. In both prior studies of this phenomenon, reading times on the first noun phrase of the coordination structure (e.g. *a large pizza*) were examined. Staub and Clifton Jr. (2006) found no influence of syntactic predictability in this region for young adults, while Warren et al. (2016) observed a predictability cost for older adults, such that they took longer to read this region of text when *either* was used. One interesting possibility is that both groups make syntactic predictions of similar strength, but for older adults this process is more taxing upon limited cognitive processing resources (e.g. see Wlotko & Federmeier, 2012), resulting in a prediction cost.

Finally, there is a further theoretical question that is worth exploring using the data collected for the current study. In their prior investigation of the use of *either* to make syntactic predictions, Warren et al. (2016) found that the benefit readers gained from *either* preceding a noun‐phrase coordination was reduced in later trials of the experiment. Such a pattern of results was likely driven by participants adapting to the fact that the experimental stimuli contained a larger proportion of coordination structures than is typical of natural language, and thus adapted to expecting a coordination structure even in the absence of the word *either* (see Fine et al., 2013, for prior evidence of syntactic adaptation in reading; however, see also Harrington Stack et al., 2018; Prasad & Linzen, 2021). This raises an interesting question in relation to whether this adaptation process occurs to the same extent in young and older adults, as well as at the same rate. As mentioned above, older adults have more accumulated language experience than young adults and thus may have stronger prior expectations for what is a common sentence structure, with these expectations being less susceptible to new experiences than those of young adults. Thus, a further question we intend to explore is the extent to which young and older adults adapt to processing the syntactic structures used in the current study.

## METHOD

### Participants

Participants were 60 young (mean age = 22.4 years; age range = 21–25 years; 13 males; mean years of formal education = 14.85; and mean hours reading per week = 12.57) and 60 older adults (mean age = 69.5 years; age range = 65–88 years; 34 males; mean years of formal education = 14.54; and mean hours reading per week = 20.35). All participants were native English speakers. All older adults and 52 of the young adults were recruited via Prolific academic (www.prolific.co), a web‐based platform for recruiting participants for online research; another 8 young adults were recruited online from our institution's undergraduate population, with these participants also completing the experiment remotely. Participants were compensated with money or course credit.

### Materials and design

**FIGURE 1:**
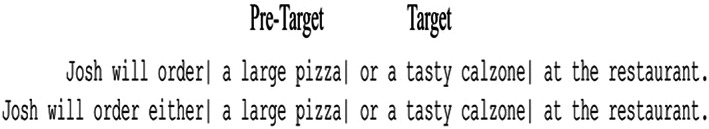
An example experimental item divided into self‐paced reading regions using the | Symbol

Participants read 32 sentences including noun‐phrase coordination structures. This structure was made predictable in half the items by including *either*, and left unpredictable in the other half through its omission; this was counterbalanced across participants. This meant our experiment followed a 2 × 2 mixed design, with a within‐participants manipulation of the presence of *either*, and a between‐participants manipulation of age group. Our stimuli comprised four presentation/analysis regions (see Figure 1); Region 1 consisted of the start of the sentence up to *either* or the preceding verb when *either* was absent; Region 2 was the first noun phrase; Region 3 was the word *or* and second noun phrase; and Region 4 the rest of the sentence. It was in Region 3 (henceforth *target region*) that we expected facilitation from *either* to occur, with a potential cost of the presence of *either* in Region 2 (henceforth *pre‐target region*). We also examined Region 4 (henceforth *post‐target region*) for any potential spill over effects. Both noun phrases typically consisted of an article, adjective and noun. Our materials are available at https://osf.io/cwyk6/?view_only=330670dc2023465b824f4e36e043bee0. These stimuli were preceded by six practice trials and intermixed with 86 filler items. Sixty‐six of these items consisted of two‐sentence texts, in which we varied whether the first sentence was presented in active or passive form, with some of these items being somewhat implausible (e.g. they involved a peasant executing a king when the opposite would be more likely). A full description of these filler items can be found in Cutter, Paterson, and Filik (2022). The remaining 20 filler items were single sentences, with half of these again including a slight implausibility (e.g. *John used an axe to chop the carrots…*).

### Procedure

Our procedure was approved by the University's Departmental Ethics Committee [F1258]. The experiment was implemented via Gorilla.sc, a web‐based client for performing behavioural experiments (Anwyl‐Irvine et al., 2020) with high temporal precision (see Bridges et al., 2020).

After providing informed consent, participants performed several tasks. Demographic information was elicited (age, hours spent reading per week, years of education and highest educational achievement) and participants performed a bot check. Next, participants completed the self‐paced reading task implemented using Gorilla's Reading Zone feature. Self‐paced reading was phrase by phrase and non‐cumulative, such that participants were initially presented with Region 1 of a sentence, while a visual mask occluded subsequent regions. The next region was revealed each time participants hit the spacebar, with the prior region replaced by the mask. Yes–no comprehension questions appeared after 40% of items within the experimental session.

Following the self‐paced reading task, participants performed a reading span and Stroop task to assess verbal working memory and inhibitory control. The purpose of these tasks was related to stimuli from another experiment in our self‐paced reading task, and are discussed further in the Appendix A.

## RESULTS

Datasets and analysis scripts are available at https://osf.io/cwyk6/?view_only=330670dc2023465b824f4e36e043bee0. Comprehension was at near ceiling in both age groups, with the mean comprehension rate being 0.98 and 0.96 among older and young adults respectively. Before data analysis, we removed reading times below 250 ms or above 5000 ms. As our target region consisted of at least three words, a minimum realistic response time of 250 ms and a maximum of 5000 ms should avoid excluding trials in which responses genuinely reflected reading processes rather than extraneous distractions.^1^ Here, we initially present a basic analysis of our data in which we simply assess the main effects of ageing and the presence of *either*, as well as the interaction between these variables. After this, we present additional analyses assessing whether older and young adults show similar adaption to the presence of certain syntactic structures across the experiment.

Mean reading times for each region are shown in Table 1. Data were log‐transformed for inferential analyses. We used Bayesian statistics to assess the effect of our manipulated variables, taking two complementary approaches. Bayesian statistics differ in focus compared to more traditional frequentist approaches. Specifically, Bayesian statistics focus on quantifying the extent to which there is either evidence for or against the existence of a particular effect, as well as how large that effect is likely to be (see Nicenboim & Vasishth, 2016, for an introduction to Bayesian statistics in language research). This contrasts with traditional, frequentist statistics in which the focus is on either the rejection or acceptance of a null hypothesis. In practice, both approaches will often (although not always) result in similar conclusions. However, the use of Bayesian statistics in the current study had the added advantage of allowing us to potentially assert that syntactic prediction is age invariant, as opposed to simply asserting no significant interaction between age and syntactic prediction.

**TABLE 1:** Mean (standard error) reading times in milliseconds across regions and conditions

	Young		Older	
	Either present	Either absent	Either present	Either absent
Pre‐target	821 (14)	780 (14)	1140 (21)	1079 (21)
Target	821 (14)	886 (16)	1215 (22)	1272 (21)
Post‐target	666 (13)	649 (11)	1177 (23)	1178 (22)

The first step in our analysis was to implement Bayesian linear mixed models, using the brms package (Bürkner, 2018) in R (R Core Team, 2020) to assess the size of any effects present in our data. Our statistical model included fixed effects for experimental condition (i.e. *either* present vs. *either* absent) and age group (i.e. young vs. older), and an interaction between these variables. We included random effects for items and participants, with all appropriate random slopes. The inclusion of random effects allows models to better account for random variance driven by both inter‐participant variability and inter‐item variability, and to guard against spurious effects. For our predictor variables, we used weakly informative priors (i.e. priors that suggested the effect would be measured on a log‐transformed reaction time scale, with very little constraints on how large the effect would be) of *Normal*(0, 1) with a regularization of 2 on the covariance matrix of random effects. Models were run with four chains of 8000 iterations each, with the first 1000 chains used as warm‐up. For each effect, we report a median effect estimate (i.e. the most likely size for the effect), the upper and lower bound of a 95% credible interval (i.e. the two values between which there is a 95% probability the parameter value lies) and *p*($\hat{b}$ > 0) or *p*($\hat{b}$ < 0) (i.e. the probability of the effect of a variable being either positive or negative respectively). We treat any effect for which *p*($\hat{b}$ > 0) is greater than .975 as being worth discussing.

In a second step in our analysis, we calculated Bayes factors (see Jeffreys, 1961; Kass & Raftery, 1995; Rouder et al., 2012) to determine whether our data were more consistent with a statistical model in which (a) age group and the presence of *either* interacted or (b) age group and the presence of *either* did not interact. Bayes factors provide a ratio of a dataset's marginal likelihood under two competing statistical models, such that they allow us to infer which model provides a better description of the processes that generated the data. The Bayes factor value represents the ratio of evidence for one model versus another. Here, we treat values above 3 as evidence for the interactive model and values below 1/3 as evidence for the additive model (see Lee & Wagenmakers, 2013, for a discussion of evidential categories in Bayes factors). Crucially, Bayes factors allow us to state evidence in favour of a null effect, and therefore to potentially conclude that there are no age differences in syntactic prediction, which is not possible using frequentist statistics. Bayes factors were calculated using the lmbf function in the BayesFactor library (Morey & Rouder, 2018), and by comparing a model including an interaction between age and predictability against one in which only main effects of these variables were included. Both models included random slopes for main effects only. We took this approach due to experience from prior work which suggested to us that the inclusion of a slope for the interaction often results in the Bayes factors overly favouring the null hypothesis.^2^ We calculated our Bayes factors with rscaleFixed set to the default value (‘medium’), essentially meaning Bayes factors were testing for evidence for/against medium‐effect sizes of our variables of interest. Implementing such complex models is susceptible to error, an estimate of which is returned by the Bayes factor package. If there was more than 1% error in the Bayes factor calculation, the model was recomputed until the error decreased adequately. Error of 1% should not affect our conclusions in any meaningful manner.

To start with our target region, in which the presence of *either* should facilitate reading, our model (Intercept = 6.83, CrI[6.76, 6.90]) revealed main effects of syntactic predictability (*b* = 0.06, CrI[0.04, 0.09], *p*($\hat{b}$ > 0) = 1) and age (*b* = 0.40, CrI[0.27, 0.52], *p*(*b* > 0) = 1), but no interaction (*b* = 0.00, CrI[−0.05, 0.05], *p*(*b* > 0) = .52). The Bayes factor comparing an interactive model with a non‐interactive model revealed strong evidence for the non‐interactive model (BF_10_ = 0.068). In our post‐target region (Intercept = 6.67, CrI[6.61, 6.73]), there was no spill‐over effect of the presence of *either* (*b* = −0.00, CrI[−0.02, 0.02], *p*($\hat{b}$ < 0) = .548), and no interaction between age group and the presence of *either* (*b* = 0.02, CrI[−0.03, 0.07], *p*($\hat{b}$ < 0) = .797, BF_10_ = 0.089), although there was an effect of age group whereby older adults took longer to read (*b* = 0.56, CrI[0.45, 0.67], *p*($\hat{b}$ > 0) = 1).

Our analyses of whether there were costs of the presence of *either* in the pre‐target region (Intercept = 6.74, CrI[6.68, 6.81]) revealed a cost of the presence of *either* (*b* = −0.06, CrI[−0.08, −0.03], *p*($\hat{b}$ < 0) = 1) and slower reading by older adults (*b* = 0.32, CrI[0.21, 0.44], *p*($\hat{b}$ > 0) = 1), but evidence against an interaction between these variables (*b* = 0.01, CrI[−0.05, 0.06], *p*($\hat{b}$ > 0) = 0.61; BF_10_ = 0.071).

A potential issue with the above analyses relates to the fact that older adults typically have longer reading times than younger adults, regardless of the difficulty of the text. This can be problematic when assessing age differences, as with slower overall reaction times, the difference between conditions typically increases, in the absence of any additional cognitive processing difficulty (see Hedge et al., 2018). In cases where the effect of an experimental manipulation is age invariant, this can result in apparent effects driven by general cognitive slowing as opposed to additional task difficulty for older adults. More saliently for the current study, it can also result in apparent age invariance when the experimental manipulation in fact had less of an effect on older compared to younger adults. One way of dealing with this issue is by z‐transforming reading times prior to analysis. To ensure that the lack of interaction between age and the presence of *either* was genuine, we z‐transformed our reading time scores and analysed these data using the same methods as above, albeit without log‐transformation. This analysis also demonstrated no interaction between age group and the presence of *either* in the pre‐target (*b* = 0.01, CrI[−0.09, 0.12], *p*($\hat{b}$ > 0) = .607, BF_10_ = 0.069), target (*b* = 0.03, CrI[−0.06, 0.13], *p*($\hat{b}$ > 0) = .756, BF_10_ = 0.075) or post‐target region (*b* = 0.04, CrI[−0.07, 0.14], *p*($\hat{b}$ > 0) = .749, BF_10_ = 0.081).

### Additional analyses

As stated above, we were also interested in assessing whether older adults and younger adults adapted to the presence of a high proportion of noun‐phrase coordination structures in our stimuli in the same manner. To test this possibility, we first constructed a statistical model in which a continuous effect of ordinal trial number interacted with the presence of *either* in the preceding sentence context. Next, we constructed two additional models, one which contained an additional two‐way interaction between age group and trial number, and one which contained a three‐way interaction among age group, trial number and the presence of *either*. To assess whether the two age groups adapted differently throughout the experiment, we compared these three models using leave‐one‐out cross‐validation (see Vehtari et al., 2017) as implemented in the loo_compare function of the loo package (Vehtari et al., 2020; version 2.3.1) in R. This procedure demonstrated that the model in which trial number only interacted with the presence of *either* had higher predictive accuracy than both the model including an interaction between age group and trial number (elpd_diff = −1.5, se_diff = 0.6) and the model including a three‐way interaction (elpd_diff = −2.9, se_diff = 0.7). Furthermore, in the model including a three‐way interaction, the model estimate for the effect suggested very little in the way of a three‐way interaction (*b* = 0.01, CrI[−0.03, 0.05], *p*($\hat{b}$ < 0) = .63). Thus, our data do not suggest that syntactic adaptation was occurring differentially in younger versus older adults in the current study. It is worth noting that, in our best performing model, there was an interaction between the presence of *either* and trial number (*b* = −0.02, CrI[−0.04, −0.00], *p*($\hat{b}$ < 0) = .977) such that readers gained less benefit from *either* as the experiment proceeded, with reading times for sentences not including *either* decreasing substantially as the experiment progressed (i.e. a decrease of 255 ms between the first and second half of the experiment) with a much smaller decrease for sentences including *either* (i.e. a decrease of 116 ms). This replicates an aspect of Warren et al.’s (2016) older adult data while extending this finding to younger adults.

## DISCUSSION

With the present experiment, we assessed whether young and older adults use the reliable cue of *either* to make syntactic predictions to a similar or different extent. Our statistical analyses suggested that both participant groups made similar use of this predictive cue, such that there was strong evidence against an interaction between age group and the presence of *either* in our Bayes factor analysis, at the target (e.g. *or a tasty calzone*), pre‐target (e.g. *a large pizza*) and post‐target region, in both log‐transformed and z‐transformed reading times. There was, however, a clear benefit of *either* at the target region and a clear cost at the pre‐target region. Thus, young and older adults made syntactic predictions of similar strength in the current study. Furthermore, processing costs that may have been related to this prediction – as measured by reading times at the pre‐target region – were equivalent for both age groups, suggesting that the formulation of syntactic predictions was not more taxing on cognitive resources for the older adults.

As discussed above, there is inconsistency in the literature regarding whether context use in language processing changes with age (Payne & Silcox, 2019), with most work focusing on lexical prediction and discourse processing. The current study extends this debate to syntactic prediction in reading and suggests that this aspect of prediction is age invariant. This finding is broadly in line with current evidence from research using the visual world paradigm to examine syntactic prediction during spoken language processing, which shows little or no age difference in these effects (Baltaretu & Chambers, 2018; Huettig & Janse, 2016). It also is consistent with several theoretical accounts as to how ageing might affect predictive processing. As mentioned in the Introduction, one such account proposes that any age differences in linguistic prediction may be due to differences in language exposure and world knowledge across the adult lifespan (Ryskin et al., 2020). Following this account, increasing world knowledge and language experience are less likely to alter syntactic predictions than lexical predictions, and so the absence of an age group difference in syntactic prediction, combined with evidence for age group differences in lexical prediction, would be compatible with this explanation.

Our findings can also be reconciled with accounts that assume reduced executive processing resources should result in reduced predictive processing in older compared to younger adults, and particularly two instantiations of this hypothesis outlined by Ryskin et al. (2020). In one, it is assumed that people with limited executive resources are less able to accurately maintain a context from which to generate predictions; while in another it is assumed that executive resources are needed to generate multiple potential predictions. In the present study, it was possible for readers to make a syntactic prediction using a relatively impoverished representation of the context (i.e. *either* present and followed by a noun phrase), and readers only needed to predict syntactic category as opposed to specific semantic features or orthographic representations. As such, it might be that syntactic prediction is age invariant as it imposes relatively low demands on executive resources. The present work may therefore help to refine these accounts, by placing a limit on the extent to which declines in executive resources might be expected to impact predictive processing. It should be noted that in the current study we took measures of participants' executive resources, in the form of a reading span test to assess working memory capacity and a Stroop test to assess inhibitory control (see Appendix A for a description of tasks and statistical analyses). Statistical analyses did not provide strong evidence that participants' performance on these tasks affected the size of the benefit that they obtained from the presence of *either*, supporting the idea that the linguistic predictions made in the current study were not particularly taxing upon executive processing resources, or at least the ones we measured.

Another question we were interested in was whether older adults' relatively greater level of language experience may alter the extent to which they adapt to a relatively high proportion of uncommon syntactic structures in our experiment. Warren et al. (2016) showed previously that such adaptation can take place, with no examination of whether this may occur more slowly or more quickly for older adults. It could, for example, be the case that due to a greater language experience older adults have stronger prior statistical knowledge about what is a typical sentence structure; meaning that in the context of an experiment, a greater amount of new experience is needed to affect expectations. On the other hand, other aspects of older adults' life experiences might result in them being able to adapt more quickly to novel linguistic environments – for example, someone aged over 65 more likely has experience of daily switches between, say, a work environment and communicating with a toddler than the average university student. As such, older adults might adapt either more quickly or more slowly than younger adults. As it turns out, there was no evidence that age affected the rate at which people adapted to the novel linguistic environment, although overall our participants did adapt. Thus, if such findings do reflect syntactic adaptation (see Harrington Stack et al., 2018, and Prasad & Linzen, 2021, for evidence to the contrary), it would seem that age does not affect this process. It should be noted that one potential criticism of the current work relates to the possibility that older adults could hypothetically have different expectations for how likely a coordination structure is in the absence of *either* compared to younger adults. The fact that both age groups showed an equivalent decrease in the difficulty of processing these structures throughout the experiment suggests that this is unlikely.

It is worth commenting briefly on our internet‐based approach to recruitment and data acquisition. This allowed us to recruit a larger than typical sample of older adults, thus increasing statistical power. This is especially important in a study observing no group differences, as our sample size (alongside the use of Bayesian statistics) allows us to confidently assert that our study represents evidence for age invariance, rather than simply lacking the power to detect group differences. It should be noted that we observed standard age effects (i.e. longer reading times by older adults) using this approach, demonstrating that our sample was similar to those used in lab‐based studies and that our methodology was sensitive enough to detect these effects.

Variation in reported effects of ageing and prediction in previous research exists at both the level of the experimental paradigm and in terms of how context is manipulated (for discussion, see Payne & Silcox, 2019). Accordingly, it will be important to assess whether effects are similar or different when using the same manipulation as the present experiment with different paradigms. Of particular interest would be whether syntactic prediction can be shown to be age invariant using event‐related potentials (ERPs), as weaker lexical prediction is often observed in older adults in studies using this method (e.g. Federmeier et al., 2010). Evidence exists that this method is sensitive to syntactic prediction. For instance, Lau et al. (2006; see also Neville et al., 1991) found that readers make strong local predictions about upcoming word categories following a possessive (e.g. *Max's*), such that violation of this prediction (e.g. *Max's of*) was associated with early ERP effects (e.g. a larger negative deflection at ~200 ms following stimulus onset following prediction violation). Such effects have not been investigated across adult age groups to date, to our knowledge, but would provide a strong test of adult age invariance in syntactic prediction.

Equally, it will be valuable to further investigate the present manipulation using eye movement measures. This would allow us to test the same predictions under more naturalistic reading conditions, and to implement methods to assess any subtle age differences in the use of syntactic prediction, which might include differences in parafoveal pre‐processing of linguistic information, as well as different reading strategies among the participants' groups (i.e. ‘risky’ reading; see Rayner et al., 2006; Zhang et al., 2022). Of particular relevance is the finding that older adults tend to make a greater number of regressions than young adults during natural reading, even for syntactically simple sentences. As such, it could be that eye movement measures that take account of re‐reading time (e.g. go‐past time) may reveal inflated effects in older adults not evident in the current investigation, especially as our manipulation partly exerts its effects by preventing readers from prematurely integrating the first noun phrase into the sentence. It is noteworthy, however, that regressions out of the region consisting of *or* and the second noun‐phrase in Staub and Clifton Jr. (2006) eye‐tracking study were rare (~4% of trials), leading us to doubt whether regressive eye‐movements are overly important for the current phenomenon.

A further concern is that, in the present study, we deliberately used manipulation of syntactic predictability which is highly reliable, in that noun‐phrase coordination was obligatory in sentences featuring *either* by the time readers encountered the first noun phrase. This represents an excellent starting point in establishing whether syntactic prediction may be age invariant, but leaves open the question as to whether less reliable cues to the upcoming syntactic structure are utilized equally by both younger and older adults. As such, future work could focus on less reliable cues to paint a fuller picture. For example, Staub and Clifton Jr. (2006) also examined the use of *either* to predict sentence coordination structures (e.g. *Either Linda bought the red car or her husband leased the green one*), in which *either* is more likely to predict this structure but with alternative continuations being possible such as a noun‐phrase coordination (e.g. *Either Linda bought the red car or the green car*). It might be that age differences are more likely in these structures.

A final issue concerns the cause of the processing cost in the pre‐target region. There are several possible explanations for this effect. First, it could provide evidence that some level of cognitive effort is required to make the syntactic prediction, with this increasing reading times at the pre‐target region, which is the first point that readers might begin construction of a coordination structure. Alternatively, the effect might be consistent with approaches that assume that processing difficulty at any one word is a product of the extent to which that word changes the reader's beliefs about the sentence contents (i.e. surprisal theory; Levy, 2008). Here, the reader would have their beliefs shifted towards a relatively unlikely syntactic structure (i.e. noun‐phrase coordination) after encountering *either*, with the cost of this change in belief impacting on processing at the pre‐target region. In this way, the differential patterns of effects we observed at pre‐target and target regions might reflect both the cost and benefit of the presence of *either*. It is notable that this pre‐target effect was present in Warren et al.’s (2016) study, which also used self‐paced reading, but not Staub and Clifton Jr. (2006) eye movement study. As such, it may be that this effect does not occur outside of self‐paced reading. Further work is therefore needed to establish the generality of the effect. A final possible cause of the effect relates to the way in which our sentences were presented using self‐paced reading. It may be that the prediction of a noun‐phrase coordination structure is so strong at the first noun phrase when *either* is present that depriving readers of a parafoveal preview of *or* by masking the next section of the text leads to additional processing disruption. Future eye movement research could test this possibility using the boundary paradigm (Rayner, 1975). Regardless of which explanation is correct, it should be noted that a cost in this earlier region supports the view that the effect of *either* across both noun phrases is driven by prediction rather than a later effect driven by ease of integration.

In sum, the present research presents novel evidence that older and younger adults can make similar use of unambiguous linguistic cues to predict upcoming syntactic structures during reading, and that, furthermore, older adults adapt to novel linguistic environments in a similar manner to younger adults. Our findings, therefore, contribute to an ongoing debate concerning ageing effects on the use of prediction in language processing by revealing that the use of syntactic prediction by skilled readers may be age invariant.

## AUTHOR CONTRIBUTIONS

**Michael G. Cutter:** Conceptualization; data curation; formal analysis; investigation; methodology; resources; software; writing ‐ original draft; writing ‐ review & editing. **Kevin B. Paterson:** Conceptualization; funding acquisition; methodology; project administration; supervision; writing – review and editing. **Ruth Filik:** Conceptualization; funding acquisition; methodology; project administration; resources; supervision; writing – review and editing.

## CONFLICT OF INTEREST

All authors declare no conflict of interest.

### OPEN RESEARCH BADGES

This article has earned Open Data and Open Materials badges. Data and materials are available at https://doi.org/10.17605/OSF.IO/CWYK6.

## Data Availability

The data that support the findings of this study and R Scripts used for data analysis are openly available in osf at https://osf.io/cwyk6/ (doi: https://doi.org/10.17605/OSF.IO/CWYK6).

## Footnotes

^1^
We also conducted an alternative analysis in which we removed data points that were 3 or more standard deviations from the mean for each age group. This made no difference to the inferential statistics reported in the paper.

^2^
In response to a reviewer's comment, we did also calculate Bayes factors for the interaction effect by comparing two models including the by‐item slope for the interaction term. These models also offered support for the null effect in all regions, and in both the log‐transformed and z‐transformed iteration of our analysis.

## APPENDIX A

In this appendix, we present some additional analyses of our data. In the experimental session in which our self‐paced reading data were gathered, we also assessed participants' inhibitory control and working memory capacity using additional tasks. These tasks were conducted primarily in relation to some filler items included with the materials for the current study, and we did not have strong *a priori* hypotheses about how these variables might affect task performance. Nonetheless, we present extra details on these tasks and their (lack of) interaction with our main experimental manipulation for the sake of completeness.

### A.1. Stroop task and inhibitory control

In each trial of the Stroop task, participants were presented with the word *red*, *blue* or *green* displayed in red, blue or green font, with the word being congruent with the colour on half of the trials (e.g. *red* shown in red) and incongruent in the other half of trials (e.g. *red* shown in green). The participants' task was to indicate what the display colour was as quickly and as accurately as possible. Participants responded by pressing one of three different keys on their keyboard, with each of these corresponding to a different display colour. Stroop performance was measured as the difference in a participant's mean correct response times to congruent trials vs. incongruent trials, with this being taken as a measure of inhibitory control.

We included a standardized form of this measure of inhibitory control in the main statistical models we constructed in order to test its effect on self‐paced reading, and whether it interacted with our main variables of interest (i.e. age group and the presence of *either*). As such, we included it in our statistical models as (1) a main effect; (2) in an interaction with the presence of *either*; (3) in separate two‐way interactions with both the presence of *either* and age group; and (4) in a three‐way interaction with the presence of *either* and age group. We tested the relative predictive performance of each model using the loo_compare function. Our model including no effect of inhibitory control performed almost equivalently to a model containing a main effect of inhibitory control (elpd_diff = −0.1, se_diff = 0.2), and better than the models allowing inhibitory control to interact with the presence of *either* (elpd_diff = −1.0, se_diff = 0.4), separately with the presence of *either* and age (elpd_diff = −1.0, se_diff = 0.4) and with both simultaneously in a three‐way interaction (elpd_diff = −1.9, se_diff = 0.4). Furthermore, an examination of the more complex models suggested little effect of inhibitory control on self‐paced reading performance.

### A.2. Reading span and working memory

In the reading span task, participants were shown a sentence that they had to judge as plausible or implausible as quickly as possible, with this sentence being followed by a target word shown for 1000 ms which participants had to memorize. Participants were shown blocks of between two and six sentence‐word combinations (with three blocks of each size), and at the end of a block asked to recall as many of the target words as possible. Plausibility judgements were provided via a key press, while words recalled were entered into text boxes at the end of a block. Our implementation of this task was based on Klaus and Schriefers (2016). Two scores were derived from the reading span task, and we examined their effects on our main experimental task separately. The first measure was the standardized speed at which people judged sentences to be plausible or implausible. The second was memory performance. To derive this measure, we calculated the proportion of words recalled in each block by each participant and took the average of all block scores for a participant as their overall memory score. It should be noted that this measure was missing for 12 older adults, with an error in the version of the experiment we deployed to our initial participants making these scores unreliable. This was fixed for the remaining participants. To be clear, this task was completed after the self‐paced reading task, and as such this change in protocol could not have altered performance in our main task.

We assessed the association between these measures and our main experimental task in the same way as for Stroop performance. To start with processing speed, all models performed very similarly to each other. Numerically, the model including a three‐way interaction between processing speed, age and condition had a better predictive performance than the other models, although this performance would not be considered reliably better, with the difference in elpd not being larger than even 2 standard errors. Specifically, the model with the three‐way interaction slightly outperformed the model with only a main effect of processing speed (elpd_diff = −0.1, se_diff = 2.3), the model with no effect of processing speed (elpd_diff = −0.2, se_diff = 2.2.), the model with only an interaction between condition and processing speed (elpd_diff = −0.5, se_diff = 2.0) and the model containing two separate two‐way interactions involving processing speed (elpd_diff = −0.6, se_diff = 1.8). This model provided weak evidence of a three‐way interaction (*b* = −0.04, CrI[−0.10, 0.01], *p*(*b* < 0) = .954), such that older adults gained about equal benefit from the presence of *either* regardless of processing speed in the reading span task, while younger adults with a fast‐processing speed gained no benefit from the presence of *either*, but those with a slower processing speed gained progressively larger benefits from the presence of *either*.

For the memory scores, we found a similar pattern, such that the model containing a three‐way interaction outperformed the other models numerically but not clearly enough to draw any firm conclusions. Specifically, the model containing the three‐way interaction was only very slightly better than the model containing two separate two‐way interactions involving memory score (elpd_diff = −0.1, se_diff = 1.1), with poorer performance for the models including no effect of memory (elpd_diff = −0.9, se_diff = 2.3), including just a main effect of memory (elpd_diff = −1.0, se_diff = 2.4) and including just an interaction between memory and the presence of *either* (elpd_diff = −1.2, se_diff = 1.8). While the model including a three‐way interaction among age, the presence of *either* and memory score did not actually suggest a notable three‐way interaction (*b* = 0.03, CrI[−0.02, 0.08], *p*($\hat{b}$ > 0) = .900), there was a clear two‐way interaction between age and memory capacity (*b* = −0.17, CrI[−0.31, −0.03], *p*($\hat{b}$ > 0) = .993). Here, there was a relatively small positive effect of memory score on reading times for the younger participants, and a large negative effect on reading times for older participants. This translated into a pattern whereby older adults with below‐average working memory took much longer to read than younger adults with an equivalent working memory score, while older adults with above‐average working memory scores read as quickly as younger adults with equivalently high working memory scores.
